# Hipertrofia Ventricular Esquerda: Um Fenótipo, Duas Hipóteses, Três Lições

**DOI:** 10.36660/abc.20210103

**Published:** 2021-11-01

**Authors:** Patrícia Rodrigues, Ana Rita Soares, Ricardo Taipa, Sofia Ferreira, Hipólito Reis

**Affiliations:** 1 Universitário do Porto EPE Centro Hospitalar Porto Portugal Centro Hospitalar Universitário do Porto EPE, Porto - Portugal

**Keywords:** Hipertrofia Ventricular Esquerda, Insuficiência Cardíaca, Cardiomiopatia Hipertrófica, Amiloidose, Neuropatias Amiloides Familiares/diagnóstico por imagem

## Histórico médico

Uma mulher de 58 anos com polineuropatia amiloidótica familiar causada pela mutação Val30Met (p.Val50Met) no gene da transtirretina (TTR) começou a apresentar sintomas neuropáticos aos 37 anos e uma biópsia da glândula salivar confirmou a deposição de amiloide TTR. Foi submetida a transplante hepático sete anos após o início dos sintomas e fazia uso de imunossupressores, com alterações neurológicas estáveis desde então. Ela também apresentava insuficiência renal crônica (estágio 3b) e um marcapasso implantado devido a doença do nó sinusal.

## História da apresentação

A paciente foi encaminhada ao Ambulatório de Cardiologia 14 anos após o transplante hepático, por apresentar dispneia progressiva e edema nos dois pés. Ao exame físico, apresentava sinais de congestão periférica e pulmonar.

Os resultados do ECG e a interrogação do marcapasso revelaram fibrilação atrial e estimulação ventricular com frequência cardíaca controlada.

**Figura 1:**
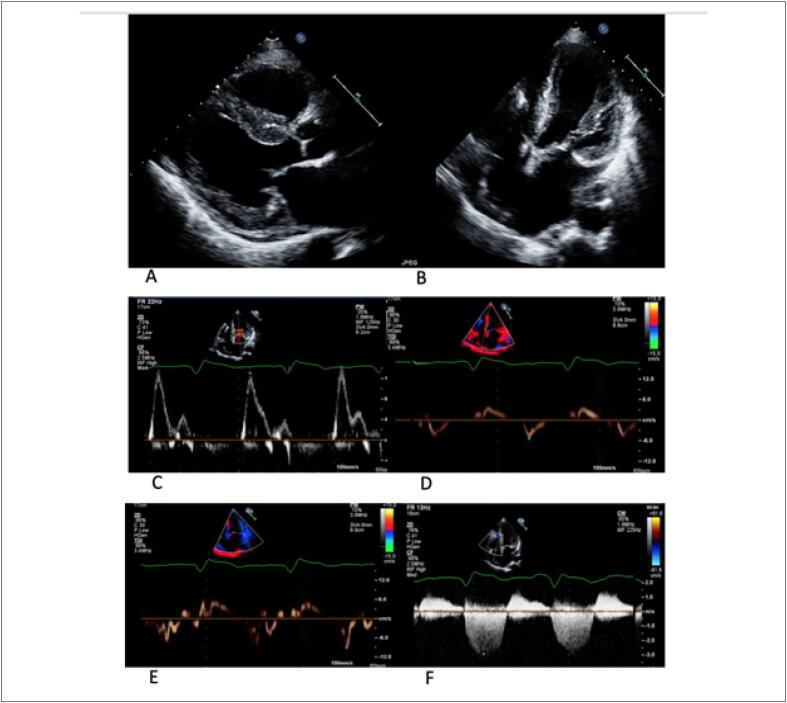
Achados da ecocardiografia transtorácica. Os painéis A (projeção paraesternal de eixo longo) e B (apical de 4 câmaras) mostram aumento da espessura da parede (máximo de 15 mm no septo interventricular basal) e átrio esquerdo levemente dilatado (volume indexado pela superfície corporal de 40 mL / m^2^). O painel C mostra uma razão E/A aumentada de 3,4. O painel D mostra uma velocidade de e' lateral de 9 cm/s e o painel E uma e' septal de 5 cm/s, dando uma média E/e' de 15. No painel F, a velocidade de regurgitação tricúspide é estimada em 2,9 m/s com Cw Doppler. Portanto, a paciente preenchia os critérios para disfunção diastólica.

O ecocardiograma transtorácico revelou hipertrofia ventricular esquerda (HVE) importante, função sistólica preservada e disfunção diastólica - Figura 1.

Iniciou anticoagulação oral e diuréticos, com melhora clínica.

## Diagnóstico diferencial

Na presença de insuficiência cardíaca com HVE, devemos primeiro considerar as condições de carga, como hipertensão ou doença valvar, que não foram observadas nesta paciente.

A cardiomiopatia hipertrófica (CMH) sarcomérica era um diagnóstico possível, que pode se apresentar com diferentes padrões de HVE, sendo a causa genética mais comum de HVE. A doença de Fabry poderia ser outra possibilidade, embora mais rara.

Contudo, nesta paciente com uma mutação conhecida, o diagnóstico mais provável era Cardiomiopatia Amiloidótica por Transtirretina (CM-ATTR). Pacientes sem cardiomiopatia significativa no momento do transplante de fígado, particularmente se sua mutação não era Val30Met, podem progredir depois, devido à deposição aumentada de proteína do tipo selvagem.^1^

## Investigações

**Figura 2:**
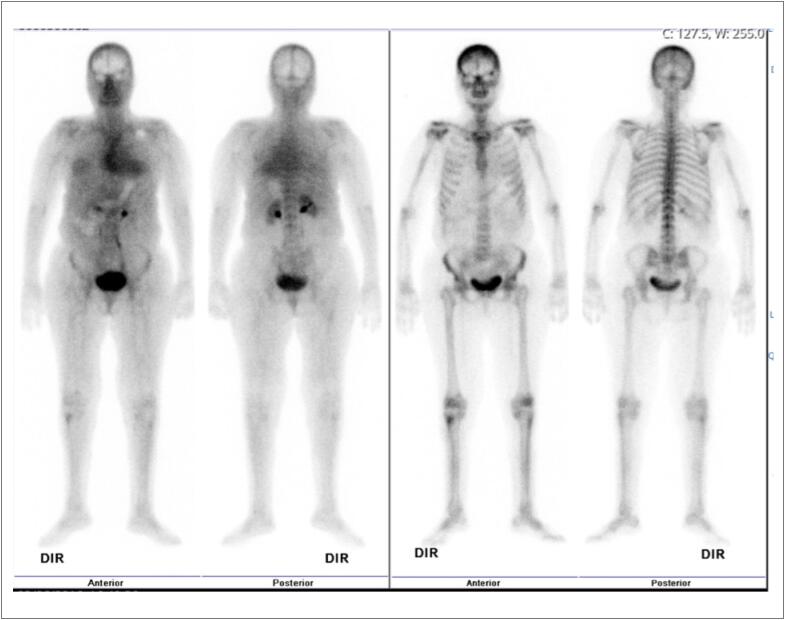
Imagens de cintilografia com Tecnécio-99m (Tc-99m) com DPD (99mTc-DPD). As imagens à esquerda foram obtidas 10 minutos após a administração do 99mTc-DPD e as imagens à direita 2 horas depois. O escore de Perugini foi zero, o que significa ausência de captação cardíaca e captação óssea normal.

Surpreendentemente, a cintilografia com Tecnécio-99m (Tc-99m) com ácido 3,3-difosfono-1,2-propanodicarboxílico (DPD) foi negativa (escore zero de Perugini) - Figura 2.

A amiloidose AL foi excluída após análise de imunofixação (urina de 24h e soro) e cadeias leves livres no soro.

A ressonância magnética cardiovascular (RMC) não foi realizada, uma vez que os eletrodos e gerador do marcapasso não eram condicionais para a RMC e a paciente era claustrofóbica. Foi solicitada biópsia endomiocárdica, que foi negativa para amiloide e não mostrou alterações significativas. Nesse ponto, outros diagnósticos foram reconsiderados para HVE.

Um estudo genético com um painel de CMH (incluindo doença de Fabry) foi solicitado e uma variante provavelmente patogênica em heterozigose foi encontrada no gene MYH7 (p.Arg783Leu). Isso nos fez questionar se o fenótipo poderia ser atribuído à CMH.

No entanto, a paciente necessitava de altas doses de diuréticos (pelo menos 120 mg de furosemida por dia para permanecer euvolêmica), embora não houvesse obstrução da via de saída do ventrículo esquerdo e era dependente de marcapasso. Revisando o ecocardiograma (Figura 1), ela apresentava um padrão de fluxo transmitral restritivo, baixas velocidades da onda S' no Doppler tecidual e ligeiro derrame pericárdico. Todos esses achados não são típicos de CMH.

**Figura 3:**
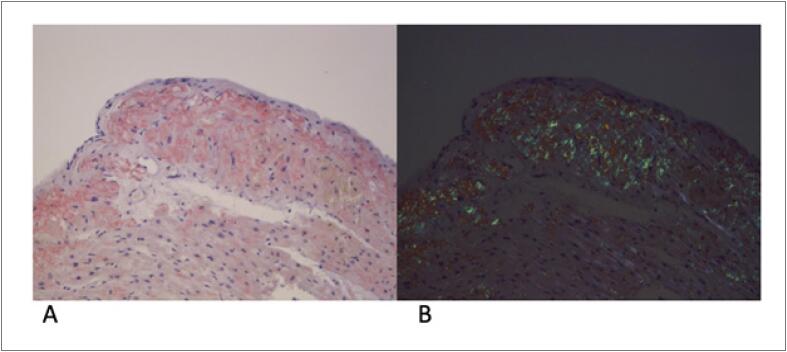
Biópsia endomiocárdica, mostrando infiltração amiloide no interstício miocárdico e particularmente no endocárdio. Coloração com Vermelho Congo (A) e coloração com Vermelho Congo sob luz polarizada (B); amplificação 200x.

Foi solicitada revisão da biópsia endomiocárdica por um patologista mais experiente, a qual na verdade mostrou infiltração amiloide grave (Figura 3).

## Discussão

Faltam critérios universalmente aceitos para o diagnóstico de cardiomiopatia amiloide, especificamente para CM-ATTR, e o algoritmo proposto por Gillmore et al.^2^ ajuda a determinar o tipo de amiloidose, mas parte de achados “sugestivos de amiloidose cardíaca”, que são bastante amplos. Uma recente declaração de posição europeia propõe um algoritmo mais claro para a suspeita e diagnóstico de amiloidose cardíaca.^3^

Geralmente, o diagnóstico requer aumento da espessura da parede ventricular (usualmente >12 mm), combinado com os resultados de exames hematológicos, cintilografia óssea e, às vezes, biópsia.

A cintilografia com ^99m^Tc-DPD mostrou excelente sensibilidade e especificidade para detectar CM-ATTR, muitas vezes dispensando a confirmação histológica,^2^ particularmente quando um escore de Perugini de 2 ou 3 (captação cardíaca moderada ou intensa) é observado.^4,5^ No entanto, mais recentemente, achados falso-negativos em imagens de radionuclídeos foram encontrados em pacientes com a mutação TTR Val30Met e início precoce de sintomas neurológicos.^6^ A causa parece estar relacionada ao fato de que esses pacientes apresentam exclusivamente fibrilas do tipo B (comprimento total), com baixa avidez por ^99m^Tc-DPD, ao contrário de pacientes com início tardio ou outras mutações, que também têm fibrilas do tipo A (truncadas).^7^ Nos primeiros casos, uma investigação adicional, incluindo biópsia endomiocárdica, pode ser necessária.

Curiosamente, nesta paciente, a biópsia endomiocárdica foi inicialmente negativa, levando-nos a explorar outros diagnósticos, a saber, CMH (como as biópsias eram muito pequenas e provenientes do ventrículo direito, a hipertrofia dos cardiomiócitos pode passar despercebida). Entretanto, devemos reconhecer que um patologista com experiência no diagnóstico de amiloidose é crucial.

Nosso grupo e vários outros descreveram o desenvolvimento de CM-ATTR anos após o transplante de fígado, não apenas em pacientes com início tardio ou mutações não-Val30Met, como relatado inicialmente, mas também em pacientes com Val30Met de início precoce. Esse fenômeno foi atribuído a mecanismos de semeadura: pequenos depósitos de fibrilas amiloides com um precursor de TTR mutado podem promover o acúmulo tardio de fibrilas de tipo selvagem. No entanto, ainda não entendemos por que esses pacientes não têm um escore positivo na cintilografia com ^99m^Tc-DPD com mais frequência, similar a pacientes com doença do tipo selvagem. Por fim, o teste genético tem sido cada vez mais útil na investigação das cardiomiopatias, mas os resultados precisam ser discutidos com cautela, pois podem ter implicações no diagnóstico e no rastreamento familiar. O conhecimento acumulado sobre cardiogênese vai esclarecer a classificação de algumas variantes. Quando uma variante patogênica ou provavelmente patogênica é encontrada, o rastreamento genético é geralmente oferecido aos membros da família.

## Conclusão

Nosso diagnóstico final foi CM-ATTR, embora os primeiros exames parecessem ter afastado essa hipótese, destacando o fato de que as biópsias endomiocárdicas são altamente dependentes do patologista e a cintilografia com ^99m^Tc-DPD pode apresentar resultados falso-negativos. Além disso, os resultados dos testes genéticos em CMH precisam ser interpretados no contexto clínico, uma vez que o achado de uma mutação, principalmente se ela não for claramente patogênica, não significa que a mesma seja a causadora do fenótipo.

Infelizmente, atualmente não há medicamentos aprovados para o tratamento da cardiomiopatia amiloide em pacientes transplantados; contudo, esperamos que isso mude em um futuro próximo.

## O que já se sabe sobre esse assunto? O que este estudo adiciona?

Este caso fornece três lições importantes:

– a identificação da causa da HVE é frequentemente negligenciada, mas buscar hipóteses diferentes e identificar a etiologia tem implicações clínicas para o paciente e sua família;
– devemos estar cientes das armadilhas da identificação de amiloide em biópsias, particularmente a importância de um patologista experiente;
– A cintilografia com ^99m^Tc-DPD também tem limitações, principalmente em pacientes com mutação Val30Met de início precoce; combinar a história clínica com os resultados de diferentes exames é fundamental para o diagnóstico da amiloidose cardíaca.
