# Supplementary material for: Hipertrofia Ventricular Esquerda: Um Fenótipo, Duas Hipóteses, Três Lições
Source: Arq Bras Cardiol. 2021 Nov 1;117(5):1056–9. [Article in Portuguese] doi: 10.36660/abc.20210103 (PMC8682104; doi:10.36660/abc.20210103)
Supplement: Translation

# Translation: Left Ventricular Hypertrophy: One Phenotype, Two Hypotheses, Three Lessons

**Authors:** Patrícia Rodrigues, Ana Rita Soares, Ricardo Taipa, Sofia Ferreira, Hipólito Reis

**Affiliations:** 1 Porto Portugal Centro Hospitalar Universitário do Porto EPE, Porto - Portugal

**Keywords:** Hypertrophy, Left Ventricular, Heart Failure, Cardiomyopathy, Hypertrophic, Amyloidosis, Amyloid Neuropathies, Familial/diagnostic imaging

## Past medical history

A 58-year-old woman with familial amyloid polyneuropathy caused by the Val30Met (p.Val50Met) mutation in the transthyretin (TTR) gene started showing neuropathic symptoms by the age of 37 and a salivary gland biopsy confirmed TTR amyloid deposition. She was submitted to liver transplantation 7 years after symptom onset and was under immunosuppressive drugs, with stable neurological changes since then. She also had chronic kidney failure (stage 3b) and a pacemaker implanted due to sick sinus syndrome.

## History of presentation

The patient was referred to the Cardiology outpatient clinic 14 years after liver transplantation, due to progressive dyspnea and bipedal edema. On physical examination, she had signs of peripheral and pulmonary congestion.

The ECG results and pacemaker interrogation revealed atrial fibrillation and ventricular pacing with controlled heart rate.

A transthoracic echocardiogram showed significant left ventricular hypertrophy (LVH), preserved systolic function and diastolic dysfunction - Figure 1.

**Figure 1:** Transthoracic echocardiogram findings. Panel A (parasternal long axis view) and B (apical 4-chamber view) show increased wall thickness (maximum 15 mm in the basal interventricular septum) and mildly dilated left atrium (body-surface indexed volume of 40 mL/m^2^). Panel C shows increased E/A ratio of 3,4. Panel D shows a lateral e' velocity of 9 cm/s and panel E a septal e' of 5 cm/s, giving an average E/e' of 15. In panel F, tricuspid regurgitation velocity is estimated at 2,9 m/s with Cw Doppler. Therefore, the patient met the criteria for diastolic dysfunction.

She was started on oral anticoagulation and diuretics, with clinical improvement.

## Differential diagnosis

In the presence of heart failure with LVH, we should first consider loading conditions such as hypertension or valvular disease, that were not observed in this patient.

Sarcomeric hypertrophic cardiomyopathy (HCM) was a possible diagnosis, which can present with different LVH patterns, being the most common genetic cause of LVH. Fabry disease could be another possibility, although rarer.

However, in this patient with a known mutation, the most likely diagnosis was

Transthyretin Amyloid Cardiomyopathy (ATTR-CM). Patients without significant cardiomyopathy at the time of liver transplantation, particularly if their mutation was not Val30Met, can subsequently progress, due to enhanced deposition of wild-type protein.^1^

## Investigations

Surprisingly, the technetium-99m 3,3-diphosphono-1,2-propanodicarboxylic acid (99mTc-DPD) scintigraphy was negative (Perugini grade of zero) - Figure 2.

**Figure 2:** Technetium-99m 3,3-diphosphono-1,2-propanodicarboxylic acid (99mTc-DPD) scintigraphy images. The pictures on the left were obtained 10 minutes after 99mTc-DPD administration and the images on the right were obtained 2 hours after. The Perugini grading score was zero, meaning no cardiac uptake and normal bone uptake.

AL amyloidosis was excluded after analyzing immunofixation (24h urine and serum) and free light chains in serum.

Cardiovascular magnetic resonance (CMR) was not performed, since the pacemaker leads and generator were not CMR conditional and the patient was claustrophobic.

An endomyocardial biopsy was requested, which was negative for amyloid and did not show significant changes. At this point, other diagnoses for LVH were reconsidered.

A genetic study with an HCM panel (including Fabry disease) was requested and a probably pathogenic variant in heterozygosity in the MYH7 gene (p.Arg783Leu) was found. This made us wonder if the phenotype could be attributed to HCM.

However, the patient needed high doses of diuretics (at least 120 mg of furosemide daily to remain euvolemic), even though no left ventricular outflow tract obstruction was seen, and she was pacemaker-dependent. Reviewing the echocardiogram (Figure 1), she had a restrictive transmitral flow pattern, low tissue Doppler S' velocities and very mild pericardial effusion. All these findings are not typical of HCM.

A review of the endomyocardial biopsy by a more experienced pathologist was requested, and it actually showed severe amyloid infiltration (Figure 3).

**Figure 3:** Endomyocardial biopsy, showing amyloid infiltration in the myocardial interstitium and particularly in the endocardium. Congo Red staining (A) and Congo Red staining under polarized light (B); amplification 200x.

## Discussion

Universally accepted criteria for diagnosing amyloid cardiomyopathy have been missing, specifically for ATTR-CM, and the algorithm proposed by Gillmore et al.2 helps to determine the type of amyloidosis, but starts from findings “suggesting cardiac amyloid”, which is quite broad. A recent European position statement proposes a more clear algorithm for the suspicion and diagnosis of cardiac amyloidosis.^3^

Usually, the diagnosis requires increased ventricular wall thickness (usually > 12 mm), combined with the results of hematologic tests, bone scintigraphy and sometimes a biopsy.

99mTc-DPD scintigraphy has shown an excellent sensitivity and specificity for detecting ATTR-CM, often precluding histological confirmation,^2^ particularly when a Perugini grade of 2 or 3 (moderate or intense cardiac uptake) is noted.^4,5^ However, more recently, false negative findings in radionuclide imaging have been found in patients with TTR Val30Met mutation and early-onset of neurological symptoms.^6^ The cause seems to be related to the fact that these patients have exclusively type B (full length) fibrils, with low avidity for 99mTc-DPD, unlike patients with late-onset or other mutations, who also have type A fibrils (truncated).^7^ In the former cases, further investigation, including endomyocardial biopsy, may be needed.

Interestingly, in this patient, the endomyocardial biopsy was initially negative, making us explore other diagnoses, namely HCM (since the biopsies were very small and from the right ventricle, cardiomyocyte hypertrophy could be missed). However, we should acknowledge that a pathologist with experience in diagnosing amyloidosis is crucial.

Our group and several others have described the development of ATTR-CM years after liver transplantation, not only in patients with late-onset or non-Val30Met mutations as initially reported, but also in early-onset Val30Met patients. This phenomenon has been attributed to seeding mechanisms: small deposits of amyloid fibrils with a mutated TTR precursor may promote late accumulation of wild-type fibrils. However, we still don't understand why these patients do not have a positive score on 99mTc-DPD scintigraphy more often, similar to patients with wild-type disease.

Finally, genetic testing has been increasingly helpful in the investigation of cardiomyopathies, but the results need to be carefully discussed, since they can have implications for the diagnosis and for family screening. The cumulative knowledge about cardiogenetics will enlighten the classification of some variants. When a pathogenic or likely pathogenic variant is found, genetic screening is usually offered to family members.

## Conclusions

Our final diagnosis was ATTR-CM, even though the first exams seemed to rule out this hypothesis, highlighting the fact that endomyocardial biopsies are highly pathologist-dependent and that 99mTc-DPD scintigraphy can have false negative results. Moreover, the results of genetic testing in HCM need to be interpreted in the clinical context, since the finding of a mutation, particularly if it is not a clearly pathogenic one, does not mean it is causing the phenotype.

Unfortunately, there are currently no drugs approved for treating amyloid cardiomyopathy in transplanted patients; however, we hope this will change in a near future.

## What is already known about this topic? What does this study add?

This case provides 3 take-home messages:

– identifying the cause of LVH is often overlooked, but pursuing different possibilities and identifying the etiology has clinical implications for the patient and the family;
– we should be aware of the pitfalls of amyloid identification in biopsies, particularly the importance of an experienced pathologist;
– 99mTc-DPD scintigraphy also has limitations, particularly in early-onset Val30Met patients; combining the medical history with the results from different exams is crucial for the diagnosis of cardiac amyloidosis.
